# ^68^Ga-Based Radiopharmaceuticals: Production and Application Relationship

**DOI:** 10.3390/molecules200712913

**Published:** 2015-07-16

**Authors:** Irina Velikyan

**Affiliations:** 1 Section of Nuclear Medicine and PET, Department of Surgical Sciences, Uppsala University, Uppsala SE-751 85, Sweden; E-Mail: irina.velikyan@akademiska.se; Tel.: +46-0-70-483-4137; Fax: +46-0-18-611-0619; 2 PET Center, Center for Medical Imaging, Uppsala University Hospital, Uppsala SE-751 85, Sweden

**Keywords:** positron emission tomography, ^68^Ga, chemistry, receptor targeting, peptide, GMP, dosimetry

## Abstract

The contribution of ^68^Ga to the promotion and expansion of clinical research and routine positron emission tomography (PET) for earlier better diagnostics and individualized medicine is considerable. The potential applications of ^68^Ga-comprising imaging agents include targeted, pre-targeted and non-targeted imaging. This review discusses the key aspects of the production of ^68^Ga and ^68^Ga-based radiopharmaceuticals in the light of the impact of regulatory requirements and endpoint pre-clinical and clinical applications.

## 1. Introduction

Development and availability of radiopharmaceuticals is a key driving force of nuclear medicine establishment and expansion. The role of ^68^Ga in the growth and worldwide spreading of clinical research and routine positron emission tomography (PET) has been proven considerable especially during last two decades. Some important features influencing such progress are the generator production of ^68^Ga, availability of commercial generators, robust labeling chemistry diversity, and potential for personalized medicine and radiotheranostics [1,2,3]. Small compounds, biological macromolecules as well as nano- and micro-particles have been successfully labeled with ^68^Ga, and the resulting agents demonstrated promising imaging capability pre-clinically and clinically [1,3,4,5,6]. In particular ^68^Ga was used for the labeling of ligands targeted to specific protein expression products such as receptors, enzymes, and antigens; small effector or hapten molecules for pre-targeted imaging; and various compounds for imaging of general biologic properties and processes such as proliferation, apoptosis, hypoxia, glycolysis, and angiogenesis. ^68^Ga is most often utilized in radiopharmaceuticals for oncology diagnostics, however its potential has also been demonstrated for imaging of myocardial perfusion, pulmonary perfusion and ventilation as well as inflammation and infection. Feasibility of non-invasive monitoring of transplantation and survival of beta cells in diabetes mellitus is one more growing application area [7,8,9]. 

**Figure 1:**
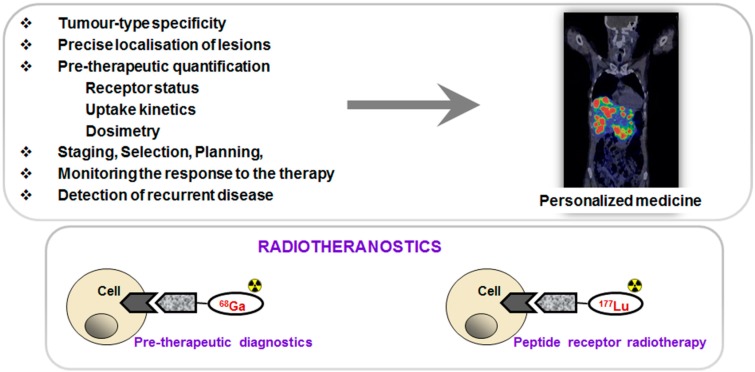
(**Upper panel**) Peptide receptor targeted imaging and radiotherapy provide personalized and thus more effective and efficient treatment of patients. (**Lower panel**) Drawing of the interaction of an agent, either imaging if labeled with ^68^Ga (**left**) or radiotherapeutic if labeled with ^177^Lu (**right**), with the cell receptor.

PET in combination with targeted imaging agents allows tumor-type specific non-invasive diagnosis with precise delineation of tumors and metastases and thus disease staging. Moreover, quantification of receptor expression, uptake kinetics and pre-therapeutic dosimetry may allow more efficient and effective treatment selection and planning as well as monitoring response to the therapy and early detection of recurrent disease resulting in personalized medicine and, in particular, radiotheranostics (Figure 1). The primary aims of the individualized patient management are to optimize therapeutic response and avoid futile treatments, minimize risks and toxicity as well as reduce cost and patient distress. Clinical intra-patient studies with variable amount of administered ^68^Ga-based imaging agents demonstrated significance of individualized patient management [10,11,12].

Such characteristics of ^68^Ga as its availability from a generator system and amenability for kit type radiopharmaceutical preparation make this radionuclide as functional as ^99m^Tc, but with additional advantages of higher sensitivity, resolution, quantification and dynamic scanning. Moreover, some therapeutic radionuclides resemble coordination chemistry of Ga(III) thus facilitating the radiotheranostic development wherein the pre-therapeutic imaging and radiotherapy are conducted with the same vector molecule exchanging the imaging and therapeutic radionuclides (Figure 1, lower panel).

A number of methods for ^68^Ga-labeling have been developed allowing choice dependent on the application objectives and logistics. Production for the clinical use can be divided into three groups: manual good manufacturing practice (GMP) production; automated GMP manufacturing; and kit type preparation. This review presents such critical aspects of ^68^Ga-radiopharmaceutical development as: generator production of ^68^Ga and its subsequent handling; essential features of labeling chemistry in relation to the endpoint biological and clinical applications; important aspects of ^68^Ga-radiopharmaceutical production process with respect to the regulatory issues. 

## 2. Characteristics and Generator Production of ^68^Ga

**Figure 2:**
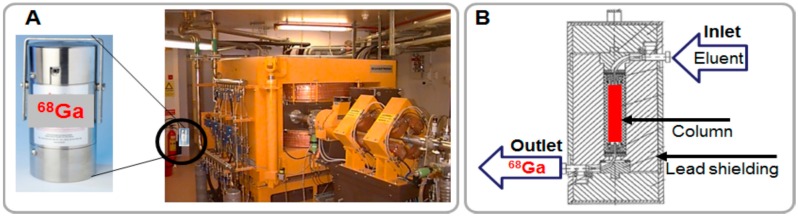
(**A**) Pictures of a ^68^Ge/^68^Ga generator and a cyclotron; (**B**) Schematic presentation of the cross section of a column-based generator.

The advantages of ^68^Ga have been presented in details previously and they are multiple with regard to both physical and chemical properties [1,3,4,6]. Those that are relevant to the aspects discussed in this review are summarized here. The high positron emission fraction (89%, E_max_: 1899 keV, E_mean_: 890 keV) and half-life of 68 min provide sufficient levels of radioactivity for high quality images while minimizing radiation dose to the patient and personnel. It requires short scanning time and allows repetitive examinations. In modern generators ^68^Ga is obtained in ionic form compatible with subsequent highly reproducible and straightforward labeling chemistry. The only oxidation state stable at physiological pH is Ga(III) providing robust labeling chemistry with ligands that can fill the octahedral coordination sphere of Ga(III) with six coordination sites. The long shelf-life generator (t_½_(^68^Ge) = 270.95 d) is simple to use and a steady source of the radionuclide for medical centers without cyclotrons or remote from distribution site. Moreover, it is a source of the enrichment of radiopharmaceutical arsenal at centers equipped with cyclotrons. As compared to cyclotron it does not require: (i) special premises with radiation shielding constructions; (ii) consumption of energy; and (iii) highly qualified personnel for running and maintaining the equipment (Figure 2A).

A generator is a self-contained system housing a parent/daughter radionuclide mixture in equilibrium. Modern commercial generators consist of a small chromatographic column situated in a shielding container (Figure 2B). ^68^Ge is produced in a high energy cyclotron from stable Ga-69 isotope (^69^Ga(p,2n)^68^Ge). Then, ^68^Ge is immobilized on a column filled with inorganic, organic or mixed matrix where it spontaneously decays to ^68^Ga (Equation (1)), which can then be extracted by an eluent. ^68^Ga decays in its turn to stable Zn(II) (Equation (2)). Thus Ge, Ga, and Zn elements populate the generator and can be found in the eluate.

(1) Ge3268+ e−10 → Ga3168+ ν

(2) Ga3168 → Zn3068+ β++ν;p →n+ β++ ν

The relation of ^68^Ge decay and ^68^Ga accumulation is described by secular equilibrium since the half-life of the ^68^Ge is over 100 times longer than that of ^68^Ga (Equation (3)).

(3) t½(Ge3268)t½(Ga3168)=5762

**Figure 3:**
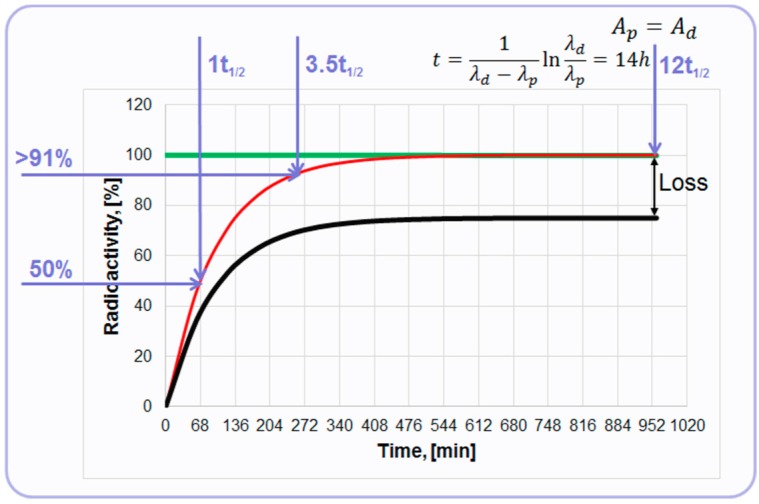
Graph of the secular equilibrium with ^68^Ge decay and ^68^Ga accumulation. The green line represents decay of ^68^Ge described by $A_{p}\left(t\right)=A_{p}\left(0\right)*\text{exp}\left(-{\text{λ}}_{p}t\right)$; the red line represents ingrowth of ^68^Ga described by $A_{d}\left(t\right)=\frac{A_{p}\left(0\right){\text{λ}}_{d}}{{\text{λ}}_{d}-{\text{λ}}_{p}}\left(\exp\left(-{\text{λ}}_{p}t\right)-\exp\left(-{\text{λ}}_{d}t\right)\right)$; and the black line represents accumulation kinetics of ^68^Ga with correction for hypothetical elution efficiency.

At the equilibrium, the radionuclides have equal radioactivities achieved at 14 h post elution (Figure 3). However, already 68 min post elution 50% of the maximum achievable radioactivity is accumulated and 4 h later it is over 91%. So, the tracer production can be performed every hour or up to three productions within one working day dependent on the generator loaded radioactivity (^68^Ge) and the age of the generator. The graph depicts a theoretical plot of ^68^Ga generation (Figure 3, red line). In reality, ^68^Ga recovery from the generator chromatographic column is less than quantitative and the proportion of ^68^Ga separated during the elution process to the theoretical value expected at the secular equilibrium is defined as elution efficiency (Figure 3, black line). The precise determination of ^68^Ga half-life time still continues reporting 67.83 min [13], 67.71 min [14], and 67.85 min [15], but for the simplicity 68 min value is used in this illustrative graph (Figure 3). It can also be mentioned that the range of the half-life values covers 62–74 min in the specification of the European Pharmacopoeia monographs [16,17].

**Figure 4:**
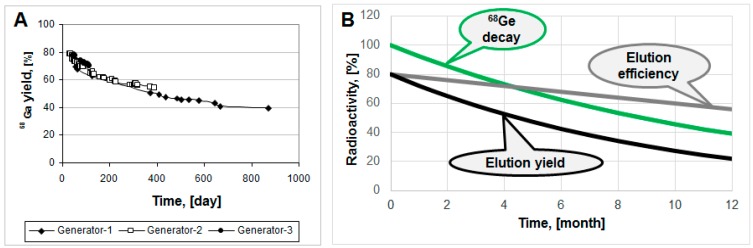
(**A**) ^68^Ga elution yield for Generator-1 over 29 months, Generator-2 over 14 months and Generator-3 over three months; (**B**) Hypothetical graphs representing ^68^Ge decay, elution efficiency and resulting non-decay corrected elution yield.

Commonly, modern ^68^Ge/^68^Ga generators demonstrate highly reproducible and robust performance, as, for example, ^68^Ga elution yield for three generator units of various age presented in Figure 4A [18]. Elution yield has both ^68^Ge-decay and elution efficiency components. The elution efficiency depends on the ^68^Ge breakthrough and column matrix, and may drop in time course, however the ^68^Ge-decay component is larger (Figure 4B).

Historically, there have been two basic Ge-Ga separation methods: liquid–liquid extraction and column technology with various eluents (alkaline, acidic or complexing agents). The column technique is most widely used with various sorbents made of inorganic, organic or mixed materials (Table 1) [1,19].

**Table 1:** Various sorbents and respective eluents used in column based ^68^Ge/^68^Ga generators.

^68^Ge/^68^Ga Generator Column Matrix	
Inorganic (Eluent)	Organic (Eluent)
SnO_2_ (1 M HCl)	*N*-methylglucamine (0.1 M HCl; 0.1 M NaOH; citrate; EDTA)
TiO_2_ (0.1 M HCl)	Pyrogallol-formaldehyde (0.3 M HCl)
CeO_2_ (0.02 M HCl)	Nanoceria-polyacrylonitrile (0.1 M HCl)
ZrO_2_ (0.1 M HCl)	
Zr-Ti ceramic (0.5 M NaOH/KOH; 4 M HCl; acetate; citrate)	
Nano-zirconia (0.01 M HCl)	

The major parameters of a generator performance are: chemical separation specificity; radiation resistance and chemical stability of the column material; eluate sterility and apyrogenecity; ^68^Ge breakthrough; eluent type; and elution profile. Most of the generators use acidic eluent since it provides cationic Ga(III) for the further direct chemistry. Inorganic column sorbents are used more widely as they are less sensitive to radiolysis. Development work continues in order to improve these characteristics and a number of commercial and in-house built generators have been introduced. Column matrixes that allow elution of ^68^Ga using several different eluents dependent of the application have been developed. Organic resin with N-methylglucamine functional groups allows elution with HCl, NaOH, citrate and EDTA dependent on the subsequent synthesis and application [20]. Highly stable nanocrystalline Zr-Ti ceramic material was developed, and the respective generator elution and in-line eluate concentration/purification was automated [21]. The elution of ^68^Ga could be performed using various eluents: basic, acidic or buffering (acetate, citrate). This generator also showed low ^68^Ge breakthrough of <10^−3^%, and the subsequent eluate purification not only further decreased ^68^Ge content (<10^−6^%) but also diminished cationic impurities. The narrow elution profile with 95% of ^68^Ga in 2 mL volume was achieved in a generator with SnO_2_ column sorbent [22]. A novel nanoceria-polyacrylonitrile-based generator provided high ^68^Ga concentration eluate and low ^68^Ge breakthrough (<10^−5^%) [23].

The modern commercial generators rely on chromatographic separation and provide advantages such as long shelf-life of 1–2 year, stable column matrixes, cationic chemical form of ^68^Ga(III) allowing subsequent versatile and direct labeling chemistry as well as reproducible and robust performance. There are a number of them with variation in the molarity of HCl eluent, metal cation content and ^68^Ge breakthrough (Table 2). This diversity is a result of over six decade journey (Table 3) from the first liquid–liquid extraction generator system [24] and simple radiopharmaceuticals for clinical application such as ^68^Ga-EDTA solution for the brain lesion imaging, ^68^Ga-citrate for bone uptake imaging, ^68^Ga-ferric oxide for bone marrow scanning, and ^68^Ga-polymetaphosphates for kidney and liver scanning [25]. Further development was directed towards the generators providing cationic ^68^Ga(III), and the first commercial one was introduced in late 1990s contributing to the blossom of the ^68^Ga-PET together with the advent of somatostatin (SST) ligands. The first generator of pharmaceutical grade appeared on the market in 2014. More generators are on their way to the market and marketing authorization acquisition.

**Table 2:** Examples of commercial ^68^Ge/^68^Ga generators.

	Eckert & Ziegler Cyclotron Co. Ltd.	Eckert & Ziegler IGG100 and IGG101 GMP; Pharm. Grade		I.D.B. Holland B.V.	Isotope Technologies Garching
	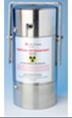	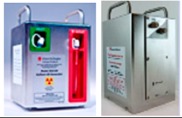		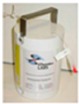	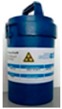
Column matrix	TiO_2_	TiO_2_		SnO_2_	SiO_2_/organic
Eluent	0.1 M HCl	0.1 M HCl		0.6 M HCl	0.05 M HCl
^68^Ge breakthrough	<0.005%	<0.001%		~0.001%	<0.005%
Eluate volume	5 mL	5 mL		6 mL	4 mL
Chemical impurity	Ga: <1 µg/mCl Ni < 1µg/mCl	Fe: <10 µg/GBq Zn: <10 µg/GBq		<10 ppm (Ga, Ge, Zn, Ti, Sn, Fe, Al, Cu)	Only Zn from decay
Weight	11.7 kg	10 kg	14 kg	26 kg	16 kg

**Table 3:** Milestones of ^68^Ge/^68^Ga generator development.

Time Period	Milestone
1950–1970	First ^68^Ge/^68^Ga generator Clinical applications: ^68^Ga-EDTA; ^68^Ga-citrate; ^68^Ga-colloid
1970–1980	Further development of ^68^Ge/^68^Ga generator: ^68^Ga(III)
1990s	Commercial generator: ^68^Ga(III)
2000s	Clinical use with advent of SST ligands
2011	GMP generators
2014	Marketing authorization

**Figure 5:**
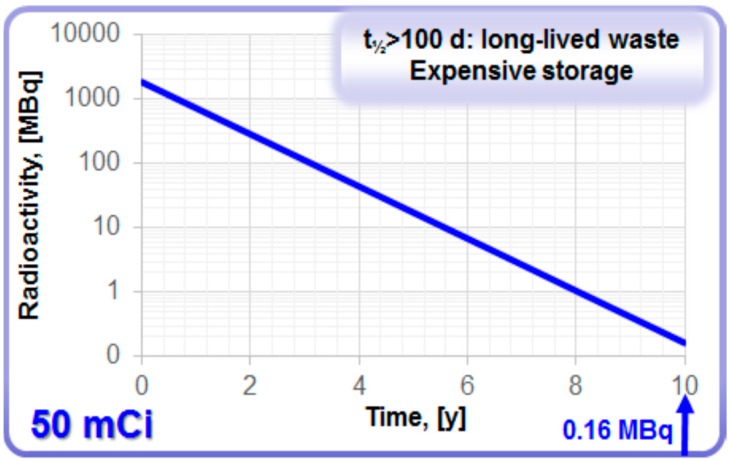
Decay of ^68^Ge in a generator with loaded radioactivity of 50 mCi.

The ^68^Ge/^68^Ga generator meets criteria of an ideal generator in terms of: efficient separation of the daughter and parent elements due to their different chemical properties; physical half-life of parent allowing rapid daughter regrowth after generator elution; stable granddaughter with no radiation dose to the patient; long shelf-life; effective shielding of the generator, minimizing radiation dose to the user and expenses of hot cells; sterile and pyrogen-free output of the generator; as well as mild and versatile chemistry of the daughter ^68^Ga amenable to automation and kit preparation. However, long shelf-life may raise concern with regard to radiolytic stability of column material, sterility of the eluate, and long-lived ^68^Ge waste management. In addition, the volatility of GeCl_4_ should be kept in mind and precautions to prevent the contamination of the surrounding must be taken. As the half-life of ^68^Ge is over 100 d, it is classified as long-lived waste. The waste and storage expenses depend on radioactivity amount and container size. It would take 10 years for the loaded radioactivity of a commonly used 50 mCi generator to decay to the amount below 1 MBq (Figure 5) that would allow regular waste. In some European countries [26] according to the regulation, the eluate of the generator must contain <10 Bq/g of ^68^Ge in order to be considered as regular waste. It has been suggested to solidify ^68^Ge in order to decrease the concentration of ^68^Ge in the bulky solution, so that the solution can be discarded as regular waste. The best-case scenario in terms of environment, sustainability and economy for the waste handling would be the re-cycling of costly ^68^Ge by the manufacturers. This would provide a problem solution on the global level. It is known that the acidic environment is not favorable to the microbiological growth and it was confirmed by loading a ^68^Ge/^68^Ga generator column intentionally with various bacteria and fungi in exhaustive amounts and following their survival during two weeks [27]. The risk of incidental microbial contamination was found very low. 

## 3. ^68^Ge/^68^Ga Generator Eluate Quality and Subsequent Labeling Chemistry

Quality and characteristics of the generator eluate including eluate volume, ^68^Ga radioactivity concentration, HCl eluent molarity, and content of metal cationic impurities influence the efficiency of ^68^Ga-labeling chemistry. Such aspects as pH, prevention of Ga(III) precipitation and colloid formation, radiolysis of vector molecules, competition of metal cations in the labeling chemistry are discussed in this section.

**Figure 6:**
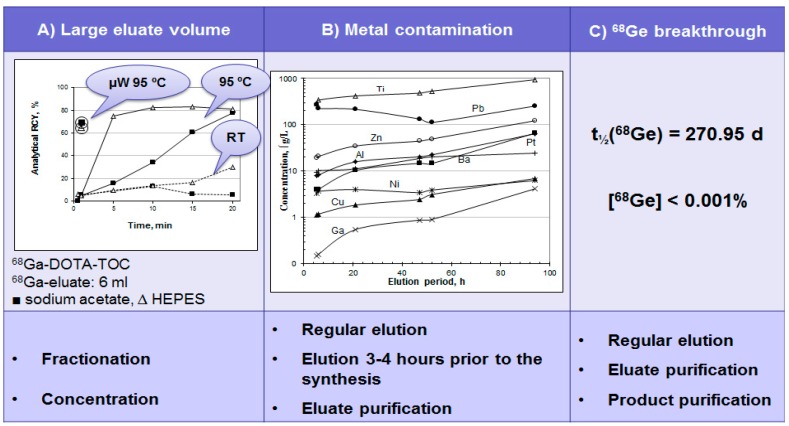
(**A**) Time course of ^68^Ga complexation reaction conducted using the full original ^68^Ga eluate (6 mL) at room temperature (**dashed line**), conventional heating in a heating block at 95 °C (**solid line**) and with microwave heating for 1 min at 90 ± 5 °C (**circled**) for two different buffer systems: ■ sodium acetate buffer, pH = 4.6, 20 nanomoles of DOTA-TOC; ∆ HEPES buffer, pH = 4.2, 20 nanomoles of DOTA-TOC; (**B**) Metal ion content in 6 mL of the generator eluate as a function of the elution time period; (**C**) ^68^Ge breakthrough with respective limit defined in European Pharmacopoeia (Ph. Eur.) monograph and methods for the reduction of the content level in the eluate and final product.

**Figure 7:**
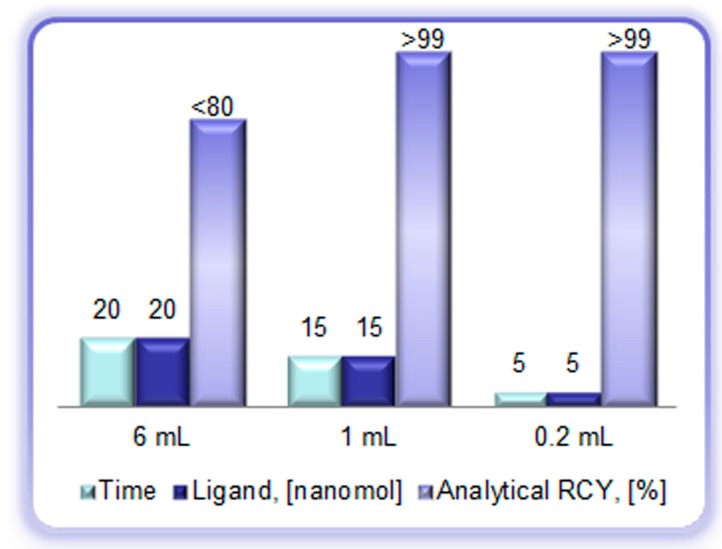
Reaction heating time (min), ligand amount (DOTA-TOC, (nanomole)), and analytical radiochemical yield (%) of the [^68^Ga]Ga-DOTA-TOC synthesis using full volume of the generator eluate (6 mL), peak fraction of the generator eluate (1 mL), and pre-concentrated/pre-purified generator eluate (0.2 mL, anion exchange method).

The disadvantages of the currently available generators are the large ^68^Ga eluate volume and consequently low ^68^Ga concentration; contamination of the eluate with long-lived parent nuclide ^68^Ge; and also presence of cationic metal ion impurities that might compete with ^68^Ga in the complexation reaction. The use of full generator eluate volume requires high ligand amount and long heating time resulting still in non-quantitative ^68^Ga incorporation (Figure 6A and Figure 7, Table 4) [18]. To overcome the drawbacks either eluate fractionation or eluate pre-concentration and pre-purification can be used (Figure 6A, Table 4). Metal cation and ^68^Ge content can be reduced by regular elution and elution prior to the synthesis as well as eluate and product purification (Figure 6B,C).

**Figure 8:**
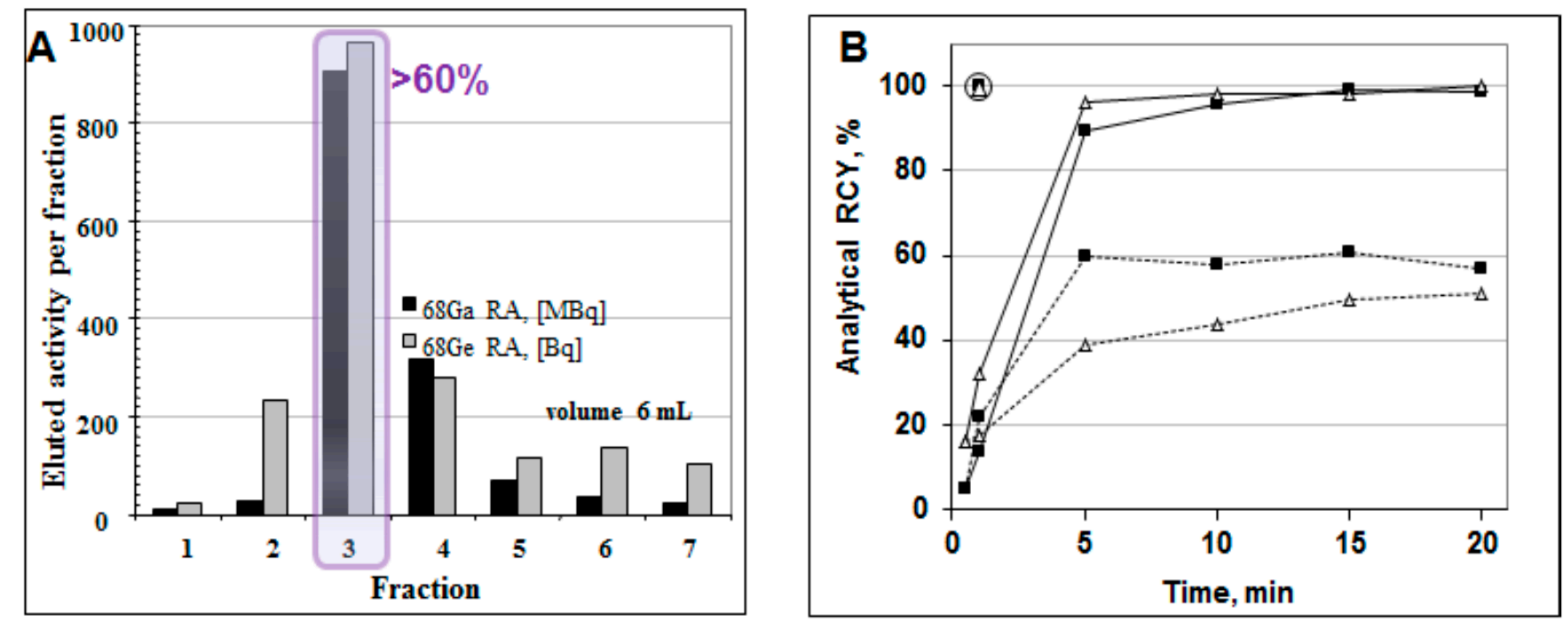
(**A**) Elution profile of the ^68^Ge/^68^Ga generator where one fraction was 1 mL (except for fraction 1 (0.3 mL) and fraction 7 (0.7 mL)), giving a total eluted volume of 6 mL. The profiles for the ^68^Ga elution and the ^68^Ge breakthrough are similar; the ^68^Ge breakthrough is ~10^−3^%. Fraction 3 (1 mL) contains over 60% of the available ^68^Ga radioactivity; (**B**) Time course of ^68^Ga complexation reaction conducted using 1 mL peak fraction of the generator eluate at room temperature (**dashed line**), conventional heating at 95 °C in a heating block (**solid line**), and with microwave heating at 90 ± 5 °C for 1 min (**circled**) for two different buffer systems: ■ sodium acetate buffer, pH = 4.6, 20 nmol of DOTA-TOC; ∆ HEPES buffer, pH = 4.2, 5 nmol of DOTA-TOC.

The collection of the top fraction decreases the eluate volume and increases ^68^Ga concentration (Figure 8A) [18,28]. Consequently, it improves the radioactivity incorporation, decreases the reaction time and required ligand amount (Figure 8B). However, it cannot remove metal cation impurities and parent ^68^Ge.

**Figure 9:**
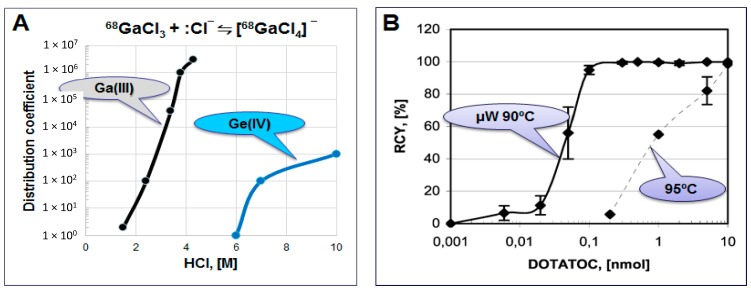
(**A**) Distribution coefficient D for the adsorption of Ga(III) and Ge(IV) chloride anions on an anion-exchange resin; (**B**) Influence of the DOTA-TOC amount on the decay-corrected radiochemical yield of the ^68^Ga complexation reaction in HEPES buffer system using the full available ^68^Ga radioactivity in 200 µL volume obtained after the pre-concentration and purification step. **Solid line**: 1 min microwave heating at 90 ± 5 °C, **dashed line**: 5 min conventional heating at 95 °C.

The methods for the eluate pre-concentration and pre-purification prior to the labeling synthesis are based on anion exchange chromatography, cation exchange chromatography or their combination (Table 4) [18,29,30,31,32,33,34,35,36]. Anion exchange method uses water for ^68^Ga recovery in 200 µL [18]. Cation exchange uses acetone/HCl mixture eluent resulting in 400 µL [34]. This method was modified in order to avoid the use of acetone that might cause formation of organic impurities [37] and appearance of acetone in the formulated product. Instead of acetone, sodium chloride [29,31] and ethanol [36] eluents were introduced as well as combined method with cation exchange for the eluate purification followed by anion exchange to eliminate the acetone [32]. However, it should be mentioned that the organic impurity formation can be avoided by storing the acetone/HCl eluent without light access and in the freezer (e.g., −20 °C). Common advantage of the pre-concentration methods is the possibility to use several tandem generators or eluates collected from several generators with the same final volume and enhanced ^68^Ga amount/concentration and generator shelf-life. Anion exchange method utilizes [^68^GaCl_4_]^−^ complex formation from ~4 M HCl medium and its absorption to the anion exchange resin (Figure 9A, Table 4). While Ge(IV) forms the anionic complex at the molarity above 5 and thus does not retain on the resin and passes through. The method allows 30–90 fold eluate volume reduction to 200 µL and is independent on the generator eluate molarity. It purifies the eluate from Ge, Al, In and Ti cations, can be accomplished within 4–6 min, and is amenable to automation. The small volume and higher concentration of ^68^Ga allows for reduced amount of the ligand and faster reaction with quantitative radioactivity incorporation and high specific radioactivity (SRA) of the tracer (Figure 7 and Figure 9B).

**Table 4:** Basic methods of ^68^Ge/^68^Ga generator eluate utilization.

Method	Eluent	Volume	Cation Impurity Reduction	^68^Ge Elimination
Full volume, 5–8 mL	H_2_O/HCl	>5000 µL	Not purified	none
Fractionation, 1 mL	H_2_O/HCl	1000 µL	Not purified	none
**Eluate Concentration and Purification**				
Anion exchange	H_2_O	200 µL	One step: Al (>99%), In (>99%), Ti (90%)	Complete
Cation exchange	Acetone/HCl	400 µL	Two steps: Zn (×10^5^), Ti (×10^2^), Fe (×10)	10^4^ fold
	NaCl/HCl	500 µL	NA	NA
	EtOH/HCl	1000 µL	Two steps: Ti (11%), Fe (×7)	400 fold
Combined cation/anion exchange	●Acetone/HCl ●H_2_O/HCl	1000 µL	NA	10^5^ fold

**Figure 10:**
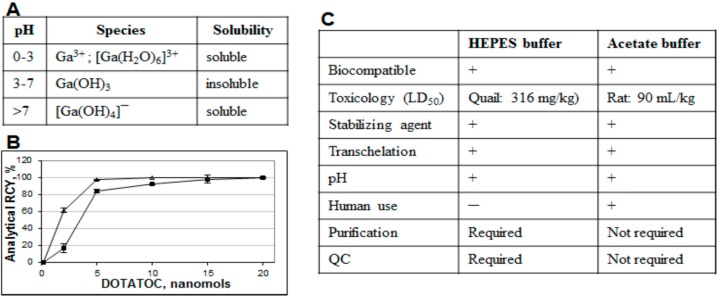
(**A**) Table showing formation of various species dependent on pH; (**B**) Influence of the buffering system (■ sodium acetate, ∆ HEPES) on the ^68^Ga radioactivity incorporation for different DOTA-TOC quantities (1 min microwave heating at 90 ± 5 °C). The reaction was conducted using the 1 mL peak fraction of the original generator eluate; (**C**) Table comparing characteristics of acetate and HEPES buffers.

Both fractionation and pre-concentration methods use hydrochloric acid in the eluent and thus require buffers for the correct pH adjustment necessary for the complexation. Moreover, weak buffer complexation capability is also essential in order to act as a stabilizing agent and prevent ^68^Ga(III) precipitation and colloid formation (Figure 10A). A number of buffering systems such as HEPES, acetate, succinate, formate, tris, and glutamate were studied with HEPES, acetate and succinate buffers demonstrating better characteristics [18,38]. In particular, HEPES and acetate buffers are both biocompatible, with no toxicity issue, providing relevant pH, and functioning as stabilizing agents. However, at lower ligand concentration, HEPES is more preferable (Figure 10B). Nevertheless from the regulatory point of view acetate has an advantage since HEPES is not approved for the human use and thus purification and additional quality control (QC) analyses are required resulting in further time and resource consumption (Figure 10C).

**Figure 11:**
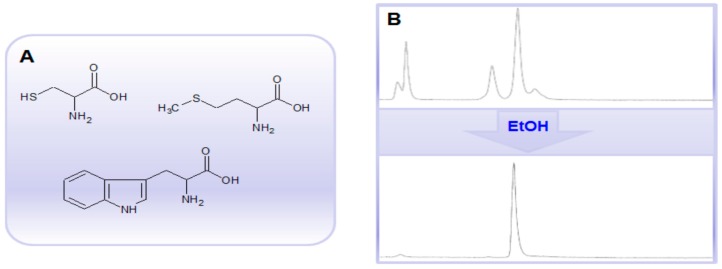
(**A**) Chemical structures of methionine, tryptophan and cysteine amino acid residues; (**B**) Radio-HPLC chromatograms of a crude ^68^Ga-labeled protein comprising ~60 amino acid residues including several tryptophan and methionine amino acid residues without (**upper panel**) and with (**lower panel**) ethanol.

The use of pre-concentration methods, especially from several generator eluates, increases ^68^Ga concentration and thus the risk of radiolysis caused by the formation of free radicals such as hydroxyl and superoxide radicals in aqueous solutions. Thus the labeling of radiosensitive compounds, e.g., peptides and proteins comprising methionine, tryptophan and cysteine amino acid residues (Figure 11A) may require presence of radical scavengers such as ascorbic acid, gentisic acid, thiols, human serum albumin, or ethanol. For example, 10%–20% of ethanol may improve the synthesis outcome considerably (Figure 11B). Moreover, ethanol is biocompatible without toxicity or immunoreactivity issues, GMP compatible, most often does not interfere with labeling reaction, and has no biological target binding capability. In addition, it can also aid the solubility of lipophilic precursors. The radical scavengers, e.g., sodium ascorbate, can also be added post synthesis to the formulated tracer. 

**Figure 12:**
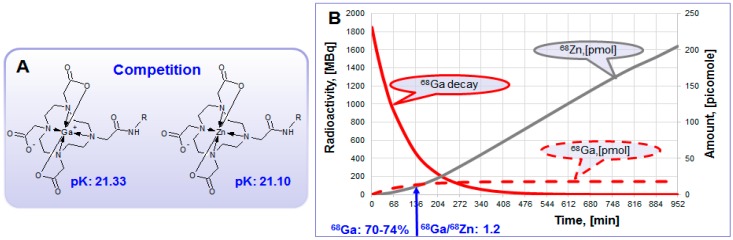
(**A**) Zn(II) forms thermodynamically stable complex with DOTA derivatives and interferes ^68^Ga-labeling reaction, especially in the excessively high concentration; (**B**) Theoretical graphs (50 mCi generator) showing ^68^Ga decay (MBq) and accumulation of radioactive ^68^Ga and stable Zn(II) in picomoles within the time frame of secular equilibrium.

The generator eluate inevitably contains a number of metal cations that may interfere with the ^68^Ga-labeling reaction (Figure 6B). Stable Zn(II) which is the product of ^68^Ga decay (Equation (2)) is rather strong competitor in complexation with DOTA-comprising agents (Figure 12A). In generator column the accumulation of Zn(II) increases continuously (Figure 12B). At the time of secular equilibrium when the maximum amount of ^68^Ga is accumulated, the amount of Zn(II) exceeds 10 times that of ^68^Ga. When generator is eluted this excess is discarded and after ~2 half-lives the ratio of ^68^Ga to Zn(II) is still over one (Figure 11B). So, regular elution and elution prior to the synthesis may allow 5.5-fold reduction of Zn(II) concentration (Figure 6B). Other solutions to overcome the reaction interference from Zn(II) could be the purification of the eluate prior to the labeling synthesis, enhanced amount of the ligand, or the use of chelators with high selectivity for Ga(III).

**Figure 13:**
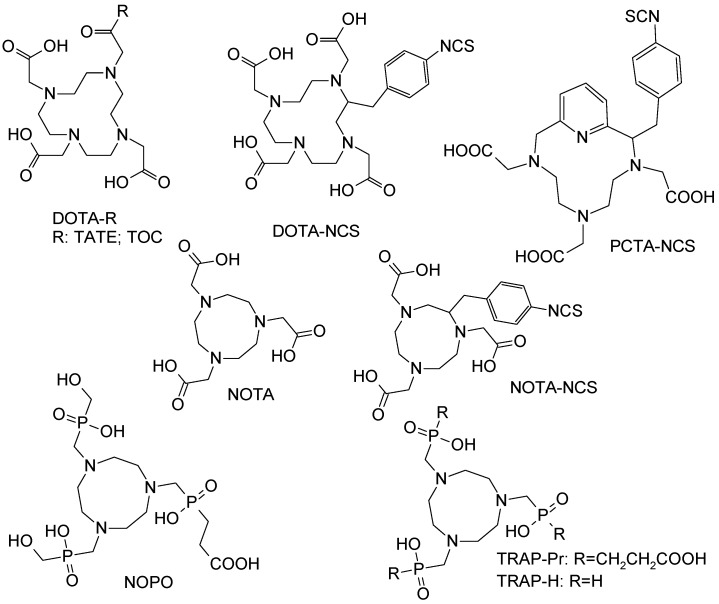
Chemical structures of example macrocyclic chelators and their derivatives that were studied in the competitive complexation reaction with various metal cations.

The eluate and reaction solutions contain other metal cations apart from Zn(II) that can compete with ^68^Ga(III), requiring higher amount of the ligand and consequently decreasing specific radioactivity. The metal cations compete with Ga(III) in different manner dependent on their chemical and physical properties such as size, charge, and surface charge density. They might form faster and more stable complexes with the same chelator or like Ti(IV) and Zr(IV) form colloids and absorb ^68^Ga(III) preventing it from the complexation. The reaction of a number of 2, 3 and 4 valent cations (Cu(II); Zn(II); Fe(II); Sn(II); Ca(II); Co(II); Ni(II); Pb(II); Al(III); Fe(III); Ga(III); In(III); Lu(III); Y(III); Yb(III); Ti(IV); Zr(IV)) relevant to the generator and labeling environment was studied with various chelators [1,39,40,41,42,43,44], especially with NOTA derivatives for which the fast labeling at room temperature was demonstrated earlier (Figure 13) [40]. Such parameters as reaction temperature, concentration, metal ion-to-ligand ratio, pH, and microwave heating mode were investigated. For DOTA derivatives, Al(III) and Ca(II) were found less critical and could be tolerated up to a concentration equal to that of the peptide bioconjugate [39,42]. The effect of the cations weakened in the following order Cu(II) > In(III) > Fe(III) > Fe(II). The radioactivity incorporation of 24% even in the presence of >1000 fold excess of Fe(III) indicated some selectivity of DOTA for Ga(III) [39]. NOTA derivatives could be radiolabeled at room temperature with over 98% yield, even in the presence of up to 10 ppm of other metal ion impurities such as Zn(II), Cu(II), Fe(III), Al(III), Sn(IV) and Ti(IV) [40,44]. Phosphinate chelators, TRAP-H, NOPO, and TRAP-Pr demonstrated efficient ^68^Ga-labeling at a wide range of pH and as acidic as pH 1 using very low amount of the ligand and thus resulting in high SRA [41,43]. In contrast to carboxylate-based chelators (NOTA and DOTA) incorporation of ^68^Ga(III) by the phosphinate chelators (TRAP-H, NOPO, and TRAP-Pr) was never entirely inhibited by the presence of Zn(II), not even at concentrations of 30 mM. Moreover, TRAP and NOPO were able to rapidly exchange coordinated Zn(II) with ^68^Ga(III), indicating high selectivity of these chelators for Ga(III). Fusarinine C, a siderophore-based chelator, also demonstrated high selectivity for ^68^Ga(III) resulting in SRA of up to 1.8 GBq/nmol [45]. Thus, the ^68^Ga-labeling efficiency in the presence of metal cation impurities can be improved by: eluting generator regularly and prior to the synthesis; pre-purification of the generator eluate by anion or cation exchange chromatography; increasing the concentration of the ligand in order to compensate for the total metal cation concentration; and employing chelators with high selectivity for Ga(III).

**Figure 14:**
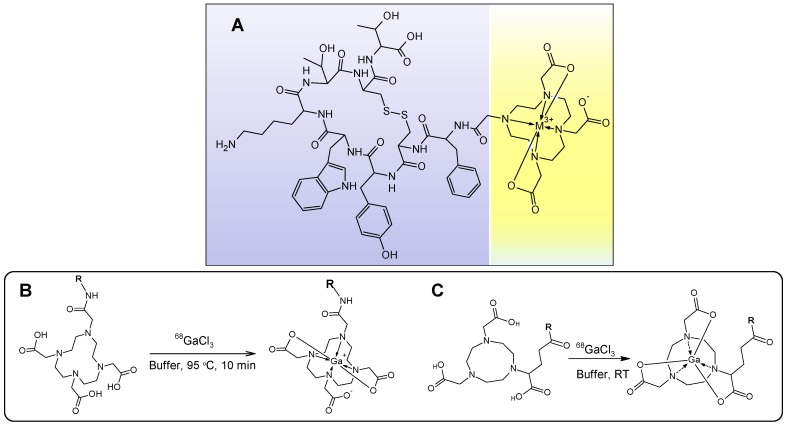
(**A**) Chemical structure of DOTA-TATE where the biologically active vector peptide (TATE, **purple background**) is conjugated to DOTA chelate moiety (**yellow background**) encaging the metal cation; (**B**,**C**) schematic presentation of ^68^Ga-labeling , respectively, with DOTA- and NOTA-based ligands, where R stands for a macromolecule such as peptide, protein, oligonucleotide, glycoprotein, antibody or low molecular weight vector that can deliver the radionuclide to the binding site.

^68^Ga has been used to label small biologically active organic molecules, biological macromolecules, complexes of variable charge and lipophilicity as well as particles [1,4,5,6]. The majority of the imaging agents are synthesized using tagging techniques wherein a vector molecule is first conjugated to a chelator moiety for the subsequent complexation with ^68^Ga (e.g., Figure 14A) [1]. The most frequently used chelators are derivatives of DOTA and NOTA that can stably complex ^68^Ga(III), respectively, at elevated and room temperature (Figure 14B,C). The latter is very important in case of temperature sensitive fragile macromolecules and also it is amenable to cold type kit production in radiopharmacy practice. The advantage of DOTA is that the same vector molecule can potentially be used both for diagnosis and radiotherapy labeling it with various radionuclides such as ^68^Ga, ^90^Y or ^177^Lu (Figure 1, lower panel).

## 4. Influence of Biological and Clinical Endpoint Applications on the Chemistry Choice 

Table 4 and Figure 7 summarize the three basic approaches of the generator eluate use with the respective key characteristics. Taking into consideration the poor labeling efficiency (Figure 6A and Figure 7), the method using full generator eluate volume directly for the labeling is basically excluded. The choice between fractionation and pre-concentration methods depends on the requirements to specific radioactivity of the imaging agent that in turn is defined by target binding site limit, pharmacological side effects or ligand cost; demand on the small solution volume and high ^68^Ga concentration; selectivity of the chelators towards ^68^Ga(III); tracer production method, e.g., automated synthesis; or kit type preparation. If high specific radioactivity is required, then the pre-purified and pre-concentrated eluate might be preferred. While fractionation method which is simpler can be used if the amount of the ligand is not limited or the chelators have high selectivity for Ga(III). If the smallest volume and highest concentration of ^68^Ga is required as, for example for the production of ^68^Ga-carbon nanoparticles [46] the anion exchange concentration might be the first choice. Kit type preparation would most probably exclude pre-concentration and pre-purification methods due to the potentially high hand dose as well as multistep protocol.

**Figure 15:**
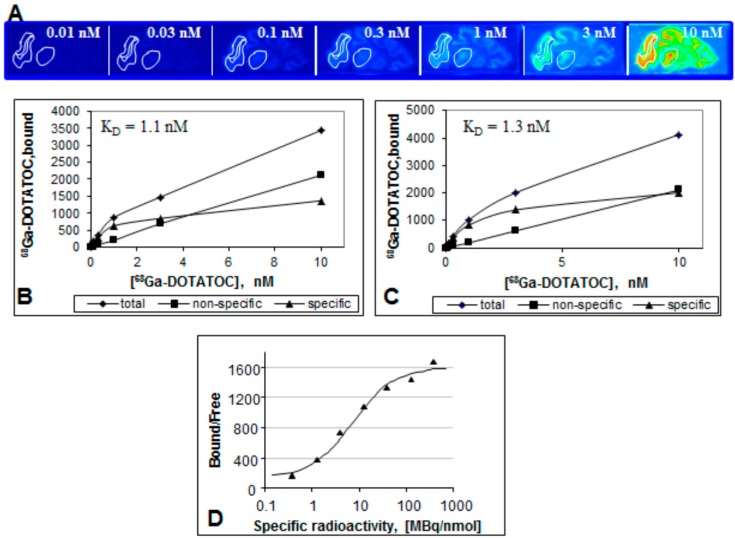
(**A**) Frozen section autoradiography showing [^68^Ga]Ga-DOTA-TOC binding to SSTR of rhesus monkey thalamus and cortex. The brain sections were incubated with different concentrations of [^68^Ga]Ga-DOTA-TOC (0.01–10 nM) for 30 min at room temperature; (**B**,**C**) Saturation of [^68^Ga]Ga-DOTA-TOC binding to SSTR of Rhesus monkey thalamus (**B**) and cortex (**C**); (**D**) The ratio of the ligand bound to the receptor and the free ligand as a function of the SRA. The data were fitted to a sigmoid two-parametric model.

SRA might be critical for endpoint biological and medical applications thus putting demand on the chemistry and such parameters as metal cation contamination, volume, and amount of the ligand. Figure 15 demonstrates an *in vitro* example where the synthesis method using anion exchange pre-concentration and pre-purification of the eluate assuring high SRA was necessary in order to obtain the saturation pattern (Figure 15B,C) and image contrast reproducibility, which could be achieved in the plateau range above SRA of 100 MBq/nmol (Figure 15D) [39]. Thus the synthesis with high SRA allowed the investigation of the influence of specific radioactivity on the binding of the tracer in frozen sections of rhesus monkey brain expressing SSTR (Figure 15A). Clear saturation allowing determination of dissociation constant, K_d_ and B_max_ could be observed (Figure 15B,C). The ratio of the amount of the receptor bound tracer to free tracer (Bound/Free (B/F)), which is also a signal to background ratio reflects contrast of an image. When presented as a function of SRA (Figure 15D) it provides important information with regard to the detection limit and reproducibility of a biological assay. In particular, the reduction of SRA corresponds to the decrease of B/F ratio and results in declined image contrast and poor detection. The most critical range of SRA values is around the inflection point where slight change of SRA may result in poor reproducibility of the image quantification. B/F becomes independent on SRA variation after it reaches the plateau. *In vivo* and *in vitro* experiments using tracers with optimized SRA would demonstrate high reproducibility and robustness.

**Figure 16:**
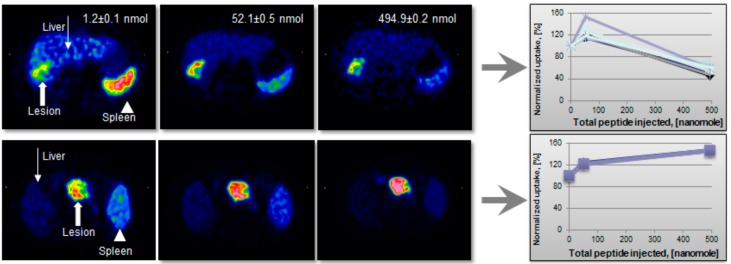
(**Upper panel**) Transaxial [^68^Ga]-DOTA-TOC-PET images of a patient with liver metastases from a colonic carcinoid who underwent three sequential PET-CT examinations. The tracer accumulation pattern in tumor tissue (**thick arrow**) increased in the second PET examination by pre-treatment with 50 µg of unlabeled octreotide but decreased again in the third examination that was proceeded by 500 µg of octreotide. (**Lower panel**) Transaxial [^68^Ga]-DOTA-TOC-PET images of a patient with a large endocrine pancreatic tumor who underwent three sequential PET-CT examinations. In contrast to the tumor uptake pattern in the other patients, as illustrated in the upper panel, the tumor accumulation (**thick arrow**) in this particular patient increased gradually over the three PET examinations.

The choice of the synthesis method and SRA optimization are also driven by the *in vivo* agent performance in terms of the target and background uptake. It can be illustrated by a study wherein three sequential examinations with gradually increasing total amount of injected peptide were performed in the same patient on the same day (Figure 16) [10]. The anion exchange pre-concentration method was necessary to employ in order to provide a wide range of SRA values. The correlation between the lesion image contrast and SRA was not linear but followed Gaussian distribution pattern. As the peptide amount increased to 50 µg the uptake in the metastases improved, while it was decreased in liver and spleen (Figure 16, upper panel). This example also stresses the importance of individualized patient management. Thus, for all but one patient the tumor and normal tissue uptake decreased after i.v. injection of 500 µg peptide (Figure 16, upper panel). For the one patient tumor uptake increased continuously (Figure 16, lower panel). Such uptake pattern variability is most probably related to the receptor density variation and would presumably indicate different therapeutic protocols.

**Figure 17:**
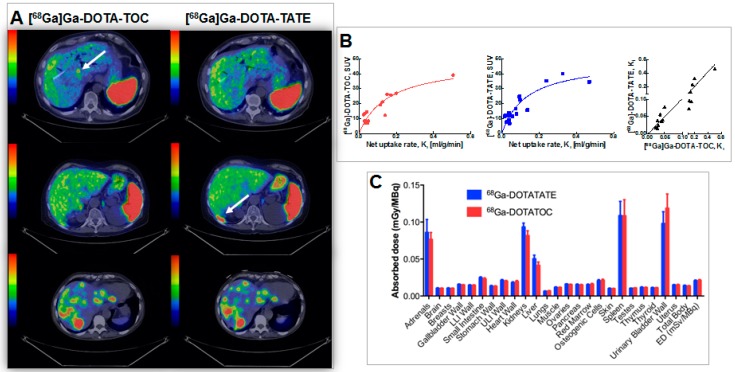
(**A**) Transaxial PET/CT fusion images of liver demonstrating cases of higher detection rate for: [^68^Ga]Ga-DOTA-TOC (**A**, **upper panel**); [^68^Ga]Ga-DOTA-TATE (**A**, **middle panel**); as well as equal detection rate (A, lower panel). Whole-body scans were conducted at 1 h p.i.. Arrows point at hepatic metastases; (**B**) SUV is presented as a function of net uptake rate K_i_ in tumors for [^68^Ga]Ga-DOTA-TOC (**red**) and [^68^Ga]-DOTA-TATE (**blue**). The solid lines are hyperbolic fits and are for visualization purposes only. Correlation between net uptake rate K_i_ at 1 h p.i. for [^68^Ga]Ga-DOTA-TOC and [^68^Ga]Ga-DOTA-TATE (**black**). The solid line is Deming regression with slope 1.06 and intercept 0.0. The axes are split in order to clarify the relationship at low uptake rates; (**C**) Absorbed doses in all organs included in OLINDA/EXM 1.1. LLI: lower large intestine; ULI: upper large intestine; ED: effective dose. Error bars indicate standard error of the mean.

The high ligand amount allowed lower SRA [10] and thus the use of simpler fractionation method that was employed in another clinical study wherein the optimized amount of the injected peptide [10] allowed high contrast and accurate comparison of two somatostatin ligand analogues ([^68^Ga]-DOTA-TOC and [^68^Ga]-DOTA-TATE, Figure 17) [47]. The identical imaging protocols and amount of administered peptide provided reliable comparison conditions. No statistically significant difference could be found in the tumor uptake of [^68^Ga]Ga-DOTA-TOC and [^68^Ga]Ga-DOTA-TATE in terms of both SUV and net uptake rate, K_i_ (Figure 17A, B). Thus, both tracers were found equally useful for staging and patient selection for peptide receptor radionuclide therapy (PRRT) in neuroendocrine tumors (NETs) with [^177^Lu]Lu-DOTA-TATE. However, the marginal difference in the healthy organ distribution and excretion may render [^68^Ga]Ga-DOTA-TATE preferable for the planning of PRRT where DOTA-TATE is used as vector. SUV did not correlate linearly with K_i_ and as such did not seem to reflect somatostatin receptor density accurately at higher SUV values, suggesting that K_i_ was the outcome measure of choice for quantification of somatostatin receptor density and assessment of treatment outcome (Figure 17B). No statistically significant difference could be observed in dosimetry estimations either (Figure 17C) [48].

Analogous results were found in another clinical study of patients affected by breast cancer using an Affibody^®^ molecule, [^68^Ga]Ga-ABY025 [11,12]. The detection rate and the image contrast were higher in the case of higher peptide amount. Again high ligand amounts allowed lower SRA and thus the use of simpler fractionation method. The patients received bolus intravenous administration of the low (LD) and high (HD) peptide content radiopharmaceuticals on two occasions one week apart. In some cases the lesion was not localized during the LD examination and the liver physiological uptake was rather high, while at HD the lesion was evident already at 1 h post injection and the image contrast increased with the time.

**Figure 18:**
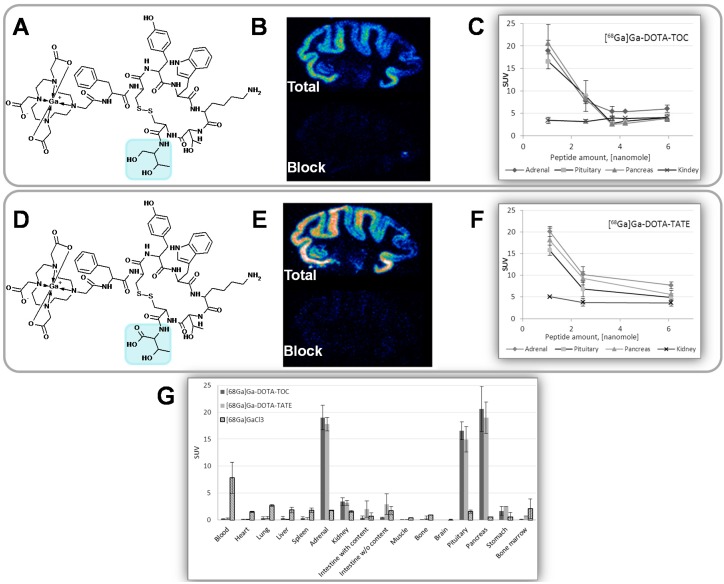
(**A**) Chemical structure of [^68^Ga]Ga-DOTA-TOC (IC_50_ = 2.5 ± 0.5; cell culture); (**B**) Rhesus monkey brain frozen section autoradiography (IC_50_ = 23.9 ± 7.9); (**C**) *In vivo* rat organ distribution for variable injected peptide amount; (**D**) Chemical structure of [^68^Ga]Ga-DOTA-TATE (IC_50_ = 0.2 ± 0.004; cell culture); (**E**) Rhesus monkey brain frozen section autoradiography (IC_50_ = 19.4 ± 8.3); (**F**) *In vivo* rat organ distribution for variable injected peptide amount; (**G**) Rat organ distribution of [^68^Ga]Ga-DOTA-TOC and [^68^Ga]Ga-DOTA-TATE 1 h post injection of 1 nmol of the peptide.

Common perception is that high affinity ligands may require tracers of high SRA. However, in the translation from cells to *in vitro* tissue and further to *in vivo* distribution, the influence of the affinity on the tracer performance may not be straightforward. The binding affinity is a parameter most often determined *in vitro* in cell cultures thus excluding *in vivo* physiological parameters. Binding affinity using frozen brain sections and biodistribution in rat were investigated for two somatostatin analogues presenting minor structural difference in C-terminal where the carboxyl group in threonine amino acid residue is exchanged to hydroxyl group (Figure 18A,D). This structural difference resulted in IC_50_ value over 10-fold difference as determined in transfected cells expressing SSTR subtype 2 [49]. However, the difference could not be observed in frozen tissue section autoradiography experiment where IC_50_ of octreotide against the two agents did not demonstrate a statistically significant difference (Figure 18B,E) [30]. There was no difference in *in vivo* and *ex vivo* biodistribution in rats in organs physiologically expressing SSTRs such as adrenals, pituitary gland and pancreas (Figure 18G). The pattern of peptide mass influence on the biodistribution was also similar for the two analogues (Figure 18C,F). As mentioned above, the clinical study also revealed no statistically significant differences in the uptake and detection rate dependent on the affinity difference [47]. In some cases [^68^Ga]Ga-DOTA-TOC detected more lesions than [^68^Ga]Ga-DOTA-TATE and vice versa (Figure 17) or the detection rate was the same. The dosimetry investigation, which is essential in order to exclude damaging effect of the radiation to the vital organs, did not reveal any statistically significant difference between the two analogues either, resulting in identical effective dose. These are illustrative examples of the complexity of the translation from the *in vitro* to *in vivo* applications. It indicated that lower values of SRA might be acceptable and thus simpler labeling techniques using fractionation method would be sufficient.

**Figure 19:**
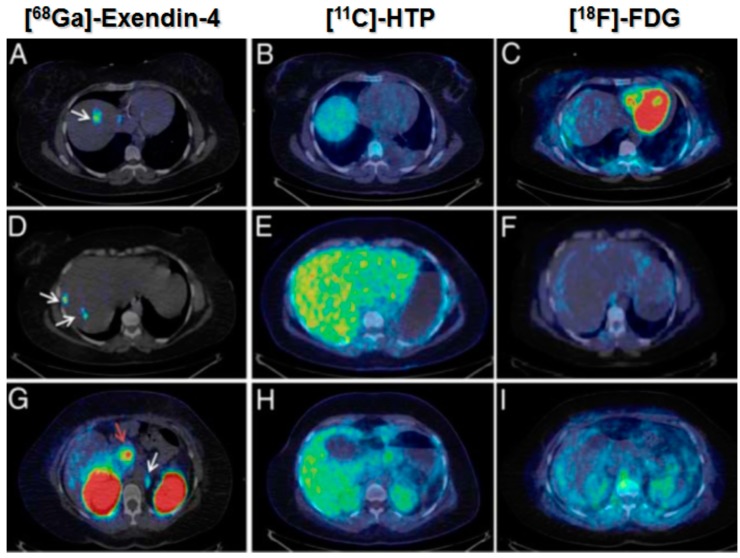
[^68^Ga]Ga-DO3A-Exendin-4/PET-CT revealed several GLP-1R positive lesions (white arrows) in the liver (**A**,**D**) and a paraortallymph node (**G**). Beta cells in normal pancreas (red arrow) have significant expression of GLP-1R and can also be visualized by this technique (**G**). No pancreatic or hepatic lesions could be detected by PET/CT using established tumor markers such as [^11^C]HTP (**B**,**E**,**H**) and [^18^F]FDG (**C**,**F**,**I**).

However, in the case of potent ligands such as Exendin-4, the amount that can be administered without induction of pharmacological effect can be very limited. Thus in the clinical examination of a patient affected by insulinoma using [^68^Ga]Ga-DO3A-Exendin-4, the maximum amount of the administered peptide was limited to 0.5 µg/kg consequently requiring higher SRA values and respective tracer production methods involving pre-concentration and pre-purification of the generator eluate. [^68^Ga]Ga-DO3A-Exendin-4/PET-CT demonstrated its uniqueness for the management of this disease group of patients. In particular, [^68^Ga]Ga-DO3A-Exendin-4/PET-CT clearly localized the lesions while conventional morphological and established physiological diagnostic techniques failed to do so (Figure 19) [50]. Thus it is important to invest resources into the tracer production development.

One more aspect that influences the choice of the imaging radionuclide labeling chemistry is the possibility of subsequent radiotherapy in the light of internal radiotheranostics. In particular the choice of the chelator is essential since any structural modification of the ligand molecule may influence its biological function and it is of outmost importance to keep the *in vivo* performance and targeting properties of the imaging and radiotherapeutic analogues as similar as possible (Figure 1). It is particularly important for targeted imaging in oncology wherein the tumor-type specific precise localization of tumors and metastases becomes possible allowing for pre-therapeutic quantification of receptor status, uptake kinetics and dosimetry and thus enabling accurate therapy selection and planning as well as monitoring response to the therapy resulting in personalized medicine (Figure 1). For example, the use of DOTA derivatives for both imaging radionuclide such as ^68^Ga(III) and therapeutic radionuclide such as ^177^Lu(III) results in relatively minor alteration in the ligand and thus might be more preferable. These radionuclides have the same charge and fit the cavity of DOTA macrocycle forming stable complexes. However, having different coordination sphere, they form complexes of different geometry, *cis*-pseudooctahedral and monocapped square antiprismatic geometry, respectively, for Ga(III) and Lu(III) [51,52]. Even minor changes in the structure of a ligand may alter its binding parameters and biodistribution pattern and thus characterization of biological activity of each specific agent must be conducted. With regard to ^68^Ga- and ^177^Lu-labeled Exendin-4 analogues the biodistribution pattern and dosimetry estimations correlated despite the differences in the radionuclide-chelator complex moiety [53,54]. 

**Figure 20:**
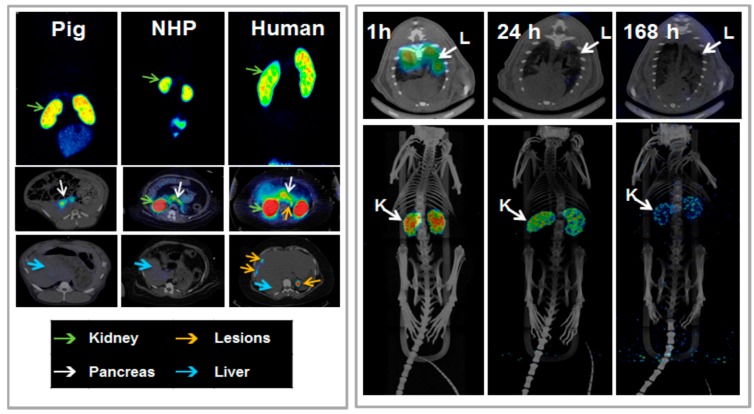
(**Left panel**) *In vivo* biodistribution of [^68^Ga]Ga-DO3A-VS-Cys^4^°-Exendin-4 as analyzed by PET-CT imaging in the pig (0.025 µg/kg; 60 min), non-human primate (NHP) (0.01 µg/kg; 90 min), and human (0.17 µg/kg; 40 min, 100 min and 120 min). The pancreas (**white arrow**) was delineated within 10 min post injection in all species. The low hepatic uptake (**blue arrow**) shows the potential for outlining insulinoma tumor metastasis (**orange arrow**, human images). The MIP coronal images demonstrate the highest uptake of the tracer in the kidneys (green arrow) in all species. (**Right panel**) Representative fused SPECT-CT images of [^177^Lu]-DO3A-VS-Cys^40^-Exendin-4 in rats at different time points. Lungs could be outlined at 1 h p.i. and showed faster clearance in later time points (**upper panel**). MIP images of whole body scan showing dominance of kidneys as excretory organ of tracer (**lower panel**).

Dosimetry investigation plays a crucial role in the radiopharmaceutical development especially in the context of internal radiotheranostics. The dosimetry estimations conducted with ^68^Ga-conterparts can presumably predict the applicability of the corresponding radiotherapeutic counterpart and thus save resources and expenses otherwise spent of the futile development of a radiotherapeutic pharmaceutical. The influence of the SRA on the biodistribution discussed in this section also applies here. However, SRA is a function of the half-life and thus direct comparison between the short-lived and long-lived radionuclides can be misleading. Therefore, it is the amount of the injected ligand that should be kept similar in the experiments. As mentioned above, [^68^Ga]Ga-DO3A-Exendin-4/PET-CT demonstrated very promising results [50] indicating that internal radiotherapy using ^177^Lu-labeled analogue might be a valuable therapeutic tool in the management of patients affected by insulinomas. However, estimation of [^68^Ga]Ga-DO3A-Exendin-4 dosimetry (Figure 20, left panel) [54] demonstrated high kidney absorbed dose that could preclude the use of [^177^Lu]Lu-DO3A-Exendin-4. This finding motivated the investigation of [^177^Lu]Lu-DO3A-Exendin-4 dosimetry using rat biodistribution for the extrapolation and estimation of human dosimetry parameters (Figure 20, right panel) [53]. The amount of the injected peptide was kept similar to that of [^68^Ga]Ga-DO3A-Exendin-4 and biodistribution pattern was comparable. The results confirmed that given the high kidney absorbed dose the amount of the acceptable administered radiation dose might be insufficient for the tumor control and thus render the treatment futile. Therefore the kidney protection and peptidase inhibition that may allow reduction of kidney absorbed dose and amplification of the tumor absorbed doses are required in order to develop [^177^Lu]Lu-DO3A-Exendin-4 for the radiotherapy.

The *in vivo* pre-clinical and clinical examples demonstrate necessity for the optimization of the SRA of the imaging/therapeutic agents in each particular case. The optimization requires wide range of SRA values and in order to provide it the highest possible SRA must be achieved using respective labeling techniques.

## 5. Regulatory Aspects

Generator is involved in the GMP production process and should comply with the requirements that would assure: product quality; patient safety; traceability of the process; reliability and robustness of the performance. Quality assurance system is necessary to ensure that quality and safety of ^68^Ga-based radiopharmaceuticals is adequate for the intended use. The qualification and validation of the performance of a chromatographic generator includes the investigation of its elution profile, elution efficiency, the extent of radionuclidic contamination of the eluate, contamination of the eluate with other metal cations and column material. To be suitable for the use in nuclear medicine, a generator must have favorable properties when these vital parameters are examined. The primary document to adhere is the European Pharmacopoeia monograph on Gallium (^68^Ga) chloride solution and Gallium (^68^Ga) edotreotide injection [16,17]. Other helpful documents are the EudraLex (Volume 4, GMP) annexes (Annex 1, Manufacture of sterile med products; Annex 3, Manufacture of radiopharmaceuticals; Annex 13, Investigational medicinal products); European Pharmacopoeia monographs (Radiopharmaceutical Preparations (0125) Ph. Eur.; Parenteral preparations (0520) Ph. Eur.; Bacterial endotoxins (20614) Ph. Eur.); Medical internal radiation dose format (MIRD); and International commission on radiological protection (ICRP). European Pharmacopoeia monographs on the compounding of radiopharmaceuticals and extemporaneous preparation of radiopharmaceuticals is in progress. The contribution of EANM, SNM and researchers around the world to the current advances in the regulatory aspects of PET radiopharmaceutical is considerable. Such issues as: regulatory documentation regarding small scale preparation of radiopharmaceuticals and the impact of the obligation to apply for manufacturing authorization or clinical trial; compliance with regulatory requirements for radiopharmaceutical production in clinical trials; quality of starting materials and final drug products/radiopharmaceuticals were thoroughly analyzed [55,56,57,58,59,60]. Guidelines on Good Radiopharmacy Practice (GRPP) [61]; patient examination protocols, interpretation and reporting of the patient examination results [62,63]; Investigational Medicinal Product Dossier; and Exploratory Investigational New Drug that reduce the demand on toxicity studies and respective cost burden as well as allow easier understanding of the regulatory requirements [64,65,66] improve professional communication and standardization. Recognition of the microdosing concept (≤100 µg or ≤30 nanomoles for peptides/proteins) [67,68,69,70] by EMEA and FDA allows validation requirements relevant to PET radiopharmaceuticals. The GMP validation expenses could further be decreased by the reduction of toxicology studies to biodistribution and dosimetry investigation specially that the latter provides more accurate and sensitive detection of distribution throughout the organs at the same time allowing monitoring adverse effects, clinical signs, clinical chemistries, hematology, histopathology, *etc.* This approach would also reduce the number of sacrificed animals adhering to the ethical norms. The work on global standardization, growth, and dissemination conducted by International Atomic Energy Agency also play essential role in the facilitation of PET introduction into clinical routine. The advent of regulatory documentation specific to PET radiopharmaceuticals introduces more clarity and improves communication between the PET community and regulatory authorities, nevertheless it should be mentioned that currently the facilitation of the entry of novel pharmaceuticals still relies mostly on magisterial and officinal preparation in combination with compassionate use under responsibility of the prescribing physician.

**Figure 21:**
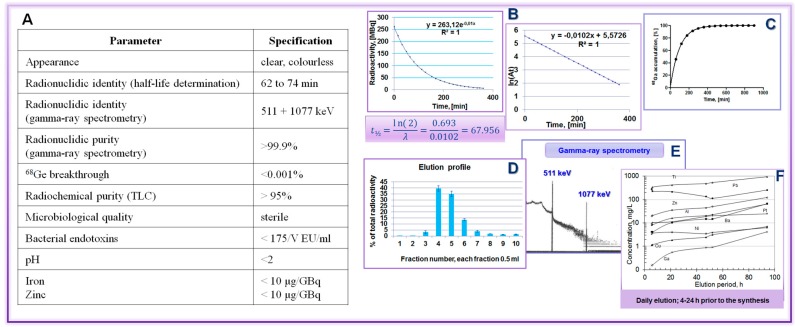
^68^Ge/^68^Ga generator validation: (**A**) Table summarizing the validation parameters and the respective specifications; (**B**) t_½_ determination; (**C**) ^68^Ga accumulation; (**D**) elution profile; (**E**) radionuclide purity; and (**F**) recommendation to elute generator prior to the synthesis.

The parameters of a ^68^Ge/^68^Ga generator that should be validated according to the specifications given in the Ph. Eur. monograph on the gallium chloride solution [16,17] are summarized in Figure 21. In addition, ^68^Ga accumulation kinetics allows choice of the generator elution and tracer production frequency. Daily elution or elution 3–4 h prior to synthesis is recommended in order to keep the metal cation impurities at lower level. In the case of a pharmaceutical grade generator the validation might be reduced to the qualification and determination of the ^68^Ge breakthrough and elution efficiency as well as a test synthesis with a validated tracer, if supported by the local quality assurance and regulation.

The content of the long-lived parent ^68^Ge in the eluate is addressed in order to assure radiation safety of the patient. The limit of 0.001% defined in the Ph. Eur. monographs was estimated assuming high and infinite accumulation of the radionuclide in sensitive organs, in particular bone marrow [71]. However, experimental evidence was necessary for the justification of the assumption. Thus, the organ distribution of ^68^Ge(IV) in rat was conducted for the extrapolation and estimation of human dosimetry parameters in order to provide experimental evidence for the determination of ^68^Ge(IV) limit [72]. While the dosimetry investigation of ^68^Ge had not been performed, the metabolism, toxicity, carcinogenicity, mutagenicity, teratogenicity as well as myopathy and nephropathy of germanium in its various chemical forms had been studied previously for various administration routs [72]. In summary, germanium as a chemical element is of low risk to man without biological function or pharmacological activity, and with fast elimination without organ accumulation. Thus with regard to radioactive ^68^Ge(IV) where the amount of the element is negligible, the safety issue is reduced to ionizing radiation and, in particular the buildup of ^68^Ga(III) (as *in vivo* generator) at the sites of deposition of ^68^Ge(IV). The dosimetry study showed that the maximum allowed administered radioactivity amount could be 645 MBq for female and 935 MBq for male, which was 35,000–50,000 higher than the level defined in the Ph. Eur. monograph. To put this in perspective, a fresh 50 mCi ^68^Ge/^68^Ga generator would allow for a breakthrough of ^68^Ge(IV) of 35 to 50% before reaching the limit doses.

**Figure 22:**
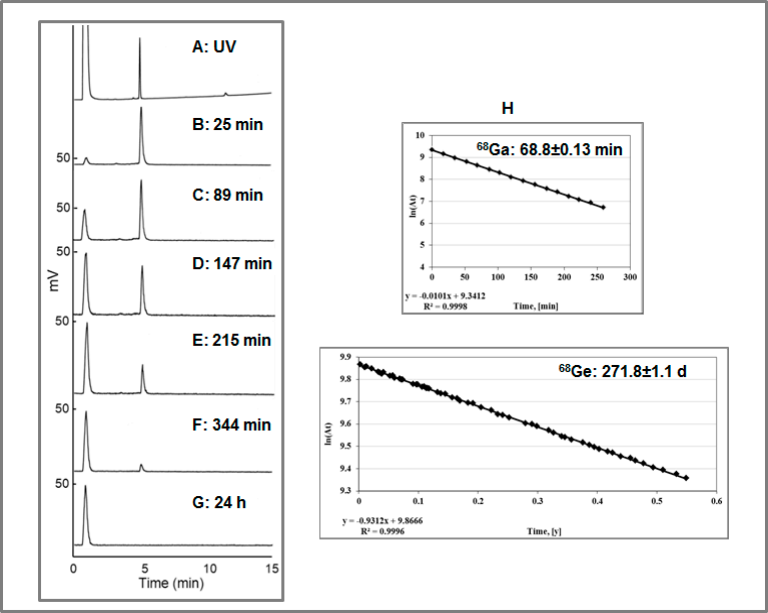
(**A**) UV-HPLC chromatogram of the authentic reference, [^Nat^Ga]-DOTA-TOC (the void signal corresponds to the buffer); (**B**–**F**) Radio-HPLC chromatograms of [^68^Ga]Ga-DOTA-TOC produced in the presence of [^68^Ge]GeCl_4_. The analysis was conducted, respectively, at 25, 89, 147, 215, and 344 min post synthesis. The signals with R_t_ of 1.0 ± 0.02 min and 4.90 ± 0.02 min correspond, respectively, to the ionic ^68^Ge(IV) and [^68^Ga]Ga-DOTA-TOC; (**G**) Radio-HPLC chromatogram taken 24 h after the production of [^68^Ga]Ga-DOTA-TOC in the presence of [^68^Ge]GeCl_4_. The signal with R_t_ of 1.0 ± 0.02 min corresponds to the ionic ^68^Ge(IV) and the signal at 4.90 ± 0.02 min corresponding to [^68^Ga]Ga-DOTA-TOC was not detected; (**H**) Determination of the t_½_ for ^68^Ga and ^68^Ge measuring respective collected chromatography fractions.

In addition, the preparation and administration of ^68^Ge(IV) was conducted in the presence and absence of [^68^Ga]Ga-DOTA-TOC simultaneous labeling synthesis. The presence of the tracer did not influence the distribution of the ^68^Ge. It was also shown that ^68^Ge(IV) was not chelated by DOTA-TOC and thus deposition of ^68^Ge in the sites of DOTA-based imaging agents accumulation is also excluded. The content of ^68^Ge and [^68^Ga]Ga-DOTA-TOC was monitored by HPLC with tandem UV and radio detectors where the signal from [^68^Ga]Ga-DOTA-TOC disappeared within 24 h while the signal from ^68^Ge remained unchanged. The respective fractions were collected and periodically measured resulting in half-life values, respectively, for ^68^Ga and ^68^Ge (Figure 22).

These results imply that the ^68^Ge(IV) limit currently recommended by monographs could be increased at least 100 times without compromising patient safety. This finding together with the availability of pharmaceutical grade generator, absence of the complexation with DOTA derivatives and knowledge that Ge(IV) does not bind to plasma proteins may facilitate the clinical introduction of kit type preparation of ^68^Ga-based imaging agents. Kit type formulation development is ongoing at both academic and industrial establishments. Several countries are working within the frame of IAEA coordinated research project (F22050) [73].

**Figure 23:**
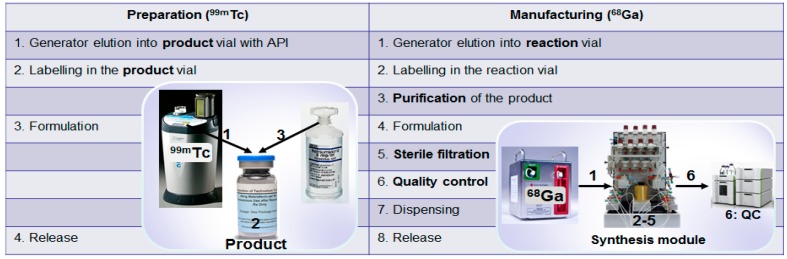
(**Left column**) Typical preparation steps of a ^99m^Tc-based registered radiopharmaceutical; (**Right column**) Basic steps of an automated manufacturing of a ^68^Ga-based radiopharmaceutical.

In general terms, a radionuclide generator is defined as a medicinal product according to the current legislation [74]. However, dissimilarities between: different radionuclide generators; the use of the generator eluate; and tracer production processes are not taken into account. For example, the eluate from ^82^Sr/^82^Rb and ^99^Mo/^99m^Tc generators enters the blood stream either directly from the generator or after the labeling reaction in the product vial (kit formulation), and thus it must be assured to be sterile, isotonic and pyrogen free (medicinal product) especially considering that sodium chloride eluent is a favorable media for microbial growth. The principle difference with regard to ^68^Ge/^68^Ga generator used in a GMP manufacturing process is that the eluate solution is removed after the labeling reaction by product purification and the final product is sterile filtered. This can be illustrated by comparison of a typical ^99m^Tc-based registered radiopharmaceutical preparation process with ^68^Ga-based imaging agent manufacturing process. The preparation under pharmaceutical practice using kit formulation technique considers direct mixing of the generator eluate with the reagents in the product vial with subsequent formulation in the same vial and release without product purification, sterile filtration and quality control (Figure 23, left column). This process results in a final radiopharmaceutical containing generator eluate components for the direct patient injection and thus the eluate quality must be assured by marketing authorization. While ^68^Ga-radiopharmaceuticals are usually manufactured under GMP environment where generator eluate either directly or after pre-purification is added to a reaction vessel, followed by the product purification, formulation, sterile filtration and release after the quality control (Figure 23, right column). Thus the final radiopharmaceutical does not contain original generator eluate solution and is sterile filtered. Essentially the manufacturing process is similar to that of cyclotron produced radionuclide-based tracers. This implies that generators with and without marketing authorization could potentially be used in such production process. The efficiency of the worldwide dissemination of ^68^Ga-radiopharmaceuticals with the patient benefit as priority would increase considerably if the essence of the manufacturing process would navigate the regulatory definition of generator produced ^68^Ga. It should be specified in each particular case dependent on the production process (kit formulation or GMP manufacturing) if it is a starting material, radionuclide precursor, active pharmaceutical ingredient or active pharmaceutical ingredient starting material. The definition would influence the choice of the generator type, namely with or without marketing authorization, potentially reducing the cost and increasing accessibility.

Radiopharmaceutical manufacturing automation provides possibility for the harmonized and standardized multicenter clinical studies that in turn may accelerate the introduction of new radiopharmaceuticals as well as their regulatory approval. Automation is important for the radioprotection, high reliability, reproducibility and robustness of the production as well as in-line traceability of the process for GMP compliance [75,76,77]. The increasing clinical demand of ^68^Ga-based tracers prompted the need for the automation. There are a number of automated synthesizers either in-house built apparatus or available on the market as standard or custom made products. Stationary tubing systems require regular cleaning and cross contamination may occur in such systems. Disposable cassette systems offer improved microbiological safety with respect to sterility and endotoxin content as well as exclude chemical cross contamination. Better cGMP compliance and simplification of the process is possible since cleaning and sanitation of the tubings, containers, and purification cartridges is avoided. The stationary tubing system on the other hand provides more flexibility and lower radiopharmaceutical production price. Fractionation, anion and cation pre-concentration methods have been automated. A number of disposable cassettes for the production of tracers for the targeted imaging of SSTR, chemokine, integrin receptors, prostate specific membrane antigene as well as inflammation visualization agent, citrate, has entered the market. 

## 6. Conclusions

Methods for the manual and automated GMP compliant production of ^68^Ga-based agents have been developed. They are based on eluate fractionation or eluate concentration and purification approaches. The choice of the labeling method depends on the endpoint pre-clinical and clinical application with respective requirements to the imaging agent characteristics. The market of generators and automated synthesis systems is expanding. The automated production improves the practicality of harmonized and standardized multicenter clinical studies facilitating the introduction of new radiopharmaceuticals. The development of kit type preparation is also feasible, although there are yet no registered ^68^Ga-radiopharmaceuticals on the market at present. Considerable advances have been made in PET radiopharmaceutical regulation and legislation still there is a number of particular questions and aspects to be addressed. Currently, understanding and support from national authorities prioritizing the benefit of patients is of outmost importance for the introduction of new radiopharmaceuticals into clinical practice.
